# Heat-Related Illness among Oregon Farmworkers

**DOI:** 10.3390/ijerph110909273

**Published:** 2014-09-05

**Authors:** Jeffrey W. Bethel, Renee Harger

**Affiliations:** College of Public Health and Human Sciences, Oregon State University, 139 Milam Hall, Corvallis, OR 97331, USA; E-Mail: renee_harger@yahoo.com

**Keywords:** heat-related illness, farmworker, Latino, migrant health

## Abstract

Farmworkers are particularly vulnerable to climate-sensitive health outcomes such as heat-related illness (HRI) given their tasks involve heavy exertion in an outdoor setting. The objectives of the study were to: (1) describe symptoms of HRI experienced by farmworkers and (2) explore factors associated with heat knowledge, level of concern regarding HRI, and comfort level taking breaks at work. Bilingual research staff conducted personal interviews of 100 farmworkers during July and August 2013. Data collected included demographics, work history and current work practices, trainings received, HRI symptoms experienced, health status, and health behaviors. Nearly 30% of participants reported experiencing ≥2 HRI symptoms during the previous work. Few participants had high level of heat knowledge (21.0%) and 15.6% of participants reported being “very concerned” about the health effects of working in hot conditions. Participants who were paid by the piece were more likely to have a high heat knowledge score and be “very concerned” about HRI but less likely to be “very comfortable” taking a break compared to workers paid by the hour than those who had not received HRI training. Results suggest several areas in which employers and agencies conducting outreach and education to the workers about HRI can change their practices including providing cooling measures and HRI training about risk factors for HRI.

## 1. Introduction

Climate change has the potential to affect human health in a variety of ways including extreme air pollution-related health effects, allergic diseases, infectious diseases, injuries, and *heat-related illnesses* (HRI) [1]. Outdoor workers have been identified as a group with increased vulnerability to climate-sensitive health outcomes such as HRI [2]. Continual exposure to high temperature and heat extremes may cause several HRI including heat rash, heat syncope (fainting), heat cramps, heat exhaustion, and heat stroke. Farmworkers are particularly at risk given their tasks involve heavy exertion in an outdoor setting. The Centers for Disease Control and Prevention reported that, between 1992–2006, 423 workers died from exposure to environmental heat (0.02 deaths per 100,000 workers) among all workers, including 68 among workers employed in crop production (0.39 deaths per 100,000 workers) [3]. Further, heat-related deaths among U.S. farmworkers are most likely to go underreported because many incidents of HRI are not identified at the time of death [4]. In addition, morbidity associated with HRI is also likely to go unreported as workers may recover on their own and not seek care [4].

Based largely on heat stress studies in athletic, military, and industrial settings, agricultural health professionals have identified strategies to prevent HRI such as hydration and elimination, rest periods, acclimatization, appropriate clothing (including headwear), worker education and employer education [4,5]. However, given the vastly different work environments in agricultural settings, barriers to HRI prevention exist [5,6]. Barriers are numerous and are rooted in culture (e.g., avoidance of certain HRI treatments, reluctance to indicate discomfort or need for water, and “machismo”), legal status (e.g., fear legal status may become known when reporting symptoms), competing health priorities (e.g., weight loss), competing workplace hazards and controls (e.g., wearing clothing to prevent chemical and ultraviolet light exposure), hydration and workplace factors (e.g., lack of hydration due to lost wages from taking breaks, perception that provided drinking water is contaminated, preference of energy drinks to improve alertness) and other workplace factors (e.g., lack of shade) [5,6]. Thus, HRI represents an area of considerable concern for health care professionals, public health practitioners and agriculture employers and employees alike.

Few studies have examined the morbidity associated with occupational HRI. Bonauto *et al*. (2007) examined the state workers’ compensation fund in Washington state from 1995–2005 and found that the HRI claim rate, 5.2 per 100,000 employees, in the agricultural, forestry, fishing and hunting industry sector was the third highest behind construction and public administration [7]. Mirabelli *et al*. (2010) collected cross-sectional survey data from 300 Latino men (n = 285) and women (n = 15) in 2009 and found that 40% of those working in extreme heat experienced symptoms of HRI at some point in their lifetime [8]. There are approximately 90,000 migrant and seasonal farmworkers in Oregon and the identification of risk factors for HRI that are specific to the agricultural setting is important in order to reduce the risk of what is postulated to be a largely preventable condition [9]. The objectives of the study were to (1) describe symptoms of HRI experienced by farmworkers and (2) explore factors associated with heat knowledge, level of concern regarding HRI, and comfort level taking breaks at work.

## 2. Methods

### 2.1. Participant Recruitment

Participants were recruited by bilingual graduate student researchers in conjunction with education and outreach staff from a local community health center. The community health center conducts annual health screenings and health education programs at nearby migrant camps. Researchers accompanied health center education and outreach workers on seven visits to four migrant camps near Cornelius, OR. The size of the camps ranged from 50 to 400 residents. At the housing camps, health center education and outreach workers referred potential participants to the interviewers who then determined eligibility and obtained informed consent into the study if the participant chose to take the survey. Participants received $20 as compensation for their participation. Eligible participants were: (1) 18 years or older; (2) farmworkers engaging in outdoor crop production at the time of the interview; (3) able to speak English or Spanish; and (4) able to provide informed consent. Oregon State University Institutional Review Board reviewed and approved the protocol to conduct research using human subjects (study identification number 5756).

### 2.2. Survey Instrument

The data collection instrument was based on a compilation of existing surveys obtained via personal communication and developed in consult with collaborators at the University of Washington [6,10]. The instrument collected information on work history, current work activities including water consumption and breaks, payment type (by piece or by hour), resources provided by employers, usual clothing and head protection worn, behaviors regarding working in hot conditions including cooling methods used, certain health conditions, and demographics. Cooling methods included use of shade structure, trees, fans, rest stations, building with air conditioning, car/truck with air conditioning, misters, wet hats, bandanas, clothing with water, water from spigot or hose, jumping into river or canal. The survey was translated by a professional translation service. To examine symptoms of HRI, participants reported whether or not they experienced any of the following symptoms during the past week while working on a hot day: skin rash/skin bumps, painful muscle cramps/spasms, dizziness/light-headedness, fainting, headache, heavy sweating, extreme weakness/fatigue, nausea/vomiting, and confusion. Using methodology by Stoecklin-Marois *et al*., a heat knowledge score was created using responses to 5 questions about heat injury prevention [10]. Scores ranged from 0 to 5 with each correct response counting as one point. “High” heat knowledge included scores of 4–5 and “low” heat knowledge included scores of less than 4. Questions assessing heat knowledge included knowledge of occupational and personal risk factors for a heat disorder and length of time to become acclimated to work after an extended absence. Level of concern about HRI was assessed using the question “How concerned are you about your health being affected by working in hot conditions”? Responses including: very concerned, a little bit concerned, not at all concerned, and no opinion. For bivariate analyses, level of concern was dichotomized into very concerned or not. Comfort level taking breaks was assessed using the question “With respect to your bosses’/contractors’ permission, how comfortable are you taking a break to drink water”? Responses including: very comfortable, somewhat comfortable, neither comfortable nor uncomfortable, a little uncomfortable, and very uncomfortable. For bivariate analyses, comfort level was dichotomized into very comfortable and all other responses collapsed into one category.

### 2.3. Data Collection and Analysis

After obtaining informed consent, bilingual interviewers completed the approximately 30 min structured personal interview in the respondents’ preferred language (English or Spanish) during 7 visits to 4 migrant camps in the months of July and August 2013. Interviews were conducted in a private area of the respondent’s choosing at the housing camp. Data from the personal interviews were entered into the Epi-Info™ and then exported to STATA (Release 12.0, STATA Corporation, College Station, TX, USA) for cleaning, coding, labeling, and statistical analysis [11,12]. We conducted univariate analysis of all variables and bivariate analysis of high heat knowledge, level of concern for HRI, and comfort level taking breaks, respectively, and key individual and work characteristics to determine prevalence estimates. Chi-square test and Fisher’s exact test were used to determine statistical association between high heat knowledge, level of concern for HRI, and comfort level taking breaks, respectively, and key individual and work characteristics. Log-binomial models and corresponding 95% confidence intervals were generated to calculate prevalence ratios. Missing data and responses of “refused to answer” and “I don’t know” were excluded from all univariate and bivariate analyses. Six variables out of 55 total variables had missing data including five variables with only one missing observation and one variable with two missing observations.

## 3. Results

A total of 100 participants completed the in-person interviews during 7 visits to 4 migrant camps. Of these, 60 were men and 40 were women ranging in age from 18 to 62 with a mean age of 31.8 (SD = 10.1) and median age of 28.5 (Table 1). Nearly all respondents (97%) were foreign-born (98% of these from Mexico) and, of these, 55.7% had been living in the U.S. for more than 10 years. Overall, 86% lived in the U.S. year-round. Eighty-four percent reported less than a high school education. Less than a quarter (24%) of respondents reported their general health as excellent or very good with 34% reporting their general health as fair or poor. Very few respondents reported being a current smoker (5%). The mean number of years that respondents have worked in agriculture in the U.S. was 8.7 and the average number of days worked in the previous week was 6.3 (Table 1). Regarding payment type, 76% of respondents reported being paid by the piece. Most participants (89.4%) picked crops and the most commonly reported crop was blueberries.

**Table 1:** Demographic and work characteristics among study participants, Oregon, 2013.

Characteristic	N = 100
Age (years); mean (SD)	31.8 (10.1)
Male gender; % (n)	60.0 (60)
Foreign-born; % (n)	97.0 (96)
Years living in U.S. ≥ 10; % (n)	55.7 (54)
Lives in U.S. all year; % (n)	86.0 (86)
Education; % (n)	
No schooling	12.0 (12)
<High school	72.0 (72)
≥High school.	16.0 (16)
Health insurance; % (n)	7.3 (7)
Self-reported general health; % (n)	
Excellent	11.0 (11)
Very good	13.0 (13)
Good	41.0 (41)
Fair/Poor	34.0 (34)
Current smoker	5.0 (5)
Number seasons worked in agriculture; mean (SD)	8.7 (6.3)
Number days worked previous 7 days; mean (SD)	6.30 (0.96)
Payment type for current job; % (n)	
Per piece	76.0 (73)
Per hour	24.0 (23)
Crops worked with previous 7 days; % (n)	
Blueberries	100.0 (100)
Apples	2.0 (2)
Pears	1.0 (1)
Cherries	2.0 (2)
Other berries	46.0 (46)
Vegetables	2.0 (2)
Other crop	7.0 (7)
Main job task previous 7 days; % (n)	
Picking	89.4 (84)
Pruning	1.1 (1)
Sorting	1.1 (1)
Other job	8.5 (8)

Sixty-four percent of respondents reported experiencing a symptom consistent with HRI during a hot day at work in the past week (Table 2). Heavy sweating (50%) and headache (24%) were the most commonly reported symptoms followed by extreme weakness/fatigue (14%) and skin rash or skin bumps (10%). One respondent reported fainting while working on a hot day during the previous week. Eleven percent of respondents reported experiencing three or more HRI symptoms. Over half (54.2%) of respondents reported receiving training about HRI in their lifetime.

**Table 2:** Frequency of heat-related illness experienced among study participants, Oregon, 2013.

Characteristic	N = 100
Ever experienced a HRI; % (n)	27.3 (27)
Ever sought treatment for HRI; % (n)	37.0 (10)
HRI symptoms experienced past week on hot day; % (n)	
Skin rash/skin bumps	10.0 (10)
Painful muscle cramps/spasms	9.0 (9)
Dizziness/light-headedness	7.0 (7)
Fainting	1.0 (1)
Headache	24.0 24)
Heavy sweating	50.0 (50)
Extreme weakness/fatigue	14.0 (14)
Nausea/vomiting	2.0 (2)
Confusion	3.0 (3)
None	36.0 (36)
Number of HRI symptoms experienced past week on hot day; % (n)	
0	36.0 (36)
1	36.0 (36)
2	17.0 (17)
≥3	11.0 (11)

Farmworkers were also asked about their practices and resources available at the worksite. Nearly half (48.3%) reported gradually increasing their work hours at the start of the season (Table 3) and most were drinking water at least once per hour in the past week (73%) as well as working relatively close (<3 min) to a water source (76%). While most respondents’ employers provided fluids at work (88.5%) and all reported drinking water (100%), many reported drinking sports drinks (69%), soda (65%), and fruit juice (41%) at work. Eleven percent also reported consuming energy drinks. Regarding access to cooling measures such as shade structures, trees, fans, rest stations, *etc*., 40% of respondents reported having no cooling measures at work. Regarding head protection, most (94%) respondents reported usually wearing a baseball cap at work during the past week and 21% indicated wearing usually wearing a wide brimmed hat at work during the past week. Regarding upper body clothing, light colored, long-sleeved shirts were most frequently reported (90%) as usual clothing worn at work during past week. Most (97%) respondents wore pants at work during the past week. According to participants’ responses, 21% scored high on the heat knowledge scale, 15.6% were “very concerned” about risk of HRI, and 45.5% were “very comfortable” taking breaks at work. There were no differences in practices and available resources by payment type (by piece or by hour).

When we generated log-binomial models to compare prevalence of high heat knowledge by key covariates, we found differences by the number of years living in the U.S. >10 years and payment type (Table 4). Specifically, a higher percentage of foreign-born respondents living in the U.S. for 10 years or more scored high on the heat knowledge score compared to foreign-born respondents living in the US for less than 10 years (PR = 1.32; 95% CI =1.08, 1.62). A higher percentage of workers paid by the piece scored high on the heat knowledge score compared to workers paid by the hour (PR = 1.29; 95% CI = 1.10, 1.52). Regarding concern level about HRI, we found differences by U.S.-birth (PR = 7.67; 95% CI = 4.52, 13.0), payment type (PR = 1.2; 95% CI =1.04, 1.39) and history of experiencing a HRI (PR = 1.42; 95% CI = 1.06, 1.89). Log-binomial models comparing prevalence of feeling “very comfortable” taking a break showed that a higher percentage of workers whose employers provided fluids were “very comfortable” taking a break compared to workers whose employers did not provide fluids (PR = 1.68; 95% CI = 1.18, 2.39). In addition, a lower percentage of workers who did not have access to cooling measures were “very comfortable” taking a break compared to workers who had access to cooling measures (PR = 0.68; 95% CI = 0.48, 0.96) and a lower percentage of workers who had a high heat knowledge score were “very comfortable” taking a break compared to workers who did not have a high heat knowledge score (PR = 0.70; 95% CI = 0.49, 0.99). Fisher’s exact tests showed differences between additional covariates and high heat knowledge, concern level about HRI, and comfort level taking breaks (Table 4).

**Table 3:** Heat-related illness knowledge and practices among study participants, Oregon, 2013.

Characteristic	N = 100
Ever received training regarding HRI; % (n)	54.2 (52)
Head protection usually worn at work during past week; % (n)	
Baseball cap	94.0 (94)
Wide brimmed hat	21.0 (21)
Other hat	2.0 (2)
Bandana	75.0 (75)
Hood from hooded sweatshirt	63.0 (63)
Clothing usually worn at work during past week; % (n)	
Light-colored short-sleeved shirt	9.0 (9)
Dark-colored short-sleeved shirt	2.0 (2)
Light-colored long-sleeved shirt	90.0 (90)
Dark-colored long-sleeved shirt	23.0 (23)
Shorts	4.0 (4)
Pants	97.0 (97)
Jacket	72.0 (72)
Gradually increased work hours at start of season; % (n)	48.3 (43)
Drank water at least once per hour past week; % (n)	73.0 (73)
Time to water source < 3 min away; % (n)	76.0 (76)
Employer provides fluids; % (n)	88.5 (85)
Time to toilet < 3 min away; % (n)	63.2(62)
No cooling measures available at current workplace; % (n)	40.0 (40)
Did not use cooling measures past week; % (n)	27.0 (27)
Heat knowledge score; % (n)	
High (4–5)	21.0 (21)
Low (<4)	79.0 (79)
Level of concern regarding risk of HRI at work; % (n)	
Very concerned	15.6 (15)
A little concerned	49.0 (47)
Not at all concerned	21.9 (21)
No opinion	13.5 (13)
Comfort level taking break to drink water; % (n)	
Very comfortable	45.5 (45)
Somewhat comfortable	32.3 (32)
Neither comfortable nor uncomfortable	10.1 (10)
A little uncomfortable	8.1 (8)
Very uncomfortable	4.0 (4)

**Table 4:** Prevalence and prevalence ratios of high heat knowledge, level of heat-related illness (HRI) concern, and comfort level taking a break by individual and work characteristics among study participants, Oregon, 2013.

Characteristic	High Heat Knowledge		Very Concerned about HRI		Very Comfortable Taking a Break	
	Prevalence	PR (95% CI)	Prevalence	PR (95% CI)	Prevalence	PR (95% CI)
*Sex*						
Female	27.5	1.0	13.2	1.0	41.0	1.0
Male	16.7	0.87 (0.70, 1.09)	17.2	1.05 (0.88, 1.24)	48.3	1.14 (0.80, 1.63)
*Country of birth*						
Foreign-born	19.8	1.0	13.0^b^	1.0	44.8	1.0
U.S.-born	66.7	2.41 (0.48, 12.0)	100.0	7.67 (4.52, 13.0)^a^	66.7	1.66 (0.33, 8.29)
*Years living in U.S.*						
<10	9.3	1.0	10.0	1.0	42.9	1.0
≥10	31.5	1.32 (1.08, 1.62)	18.9	1.11 (0.94, 1.31)	46.3	1.06 (0.74, 1.53)
*Lives in U.S. all year*						
No	20.9 ^b^	1.0	0.0	1.0	42.9	1.0
Yes	21.4	0.99 (0.74, 1.33)	18.3	---	45.9	1.06 (0.64, 1.73)
*Education*						
No schooling	8.3	1.0	8.3	1.0	41.2	1.0
<High school	22.2	1.18 (0.95, 1.45)	17.7	1.11 (0.91, 1.36)	43.7	1.04 (0.62, 1.74)
≥High school	25.0	1.22 (0.88, 1.71)	12.5	1.50 (0.15, 14.7) ^a^	56.3	1.33 (0.64, 2.78)
*Health insurance*						
No	20.2	1.0	12.9	1.0	40.9^b^	1.0
Yes	42.9	1.40 (0.30, 1.09)	28.6	1.22 (0.76, 1.96)	85.7	4.14 (0.67, 25.6)
*Payment type for current job*						
Per hour	4.4 ^b^	1.0	4.4	1.0	52.2	1.0
Per piece	26.0	1.29 (1.10, 1.52)	20.3	1.2 (1.04, 1.39)	44.4	0.86 (0.54, 1.38)
*Ever received training regarding HRI*						
No	17.3	1.0	14.0	1.0	40.9	1.0
Yes	25.0	0.91 (0.73, 1.12)	13.6	1.03 (0.37, 2.83) ^a^	52.9	1.26 (0.86, 1.84)
*Gradually increased work hours at start of season*						
No	23.9	1.0	17.4	1.0	45.7	1.0
Yes	20.9	0.96 (0.77, 1.20)	12.2	0.70 (0.25, 1.97) ^a^	50.0	1.09 (0.73, 1.62)
*Drank water at least once per hour past week*						
No	25.9	1.0	26.9	1.0	33.3	1.0
Yes	19.2	0.92 (0.71, 1.18)	11.4	0.42 (0.17, 1.05) ^a^	50.0	1.33 (0.94, 1.90)
*Time to water source < 3 min away*						
No	29.2	1.0	21.7	1.0	39.1	1.0
Yes	18.4	0.87 (0.66, 1.15)	13.7	0.63 (0.24, 1.66) ^a^	47.4	1.16 (0.78, 1.71)
*Employer provides fluids*						
No	0.0	1.0	9.1	1.0	18.2	1.0
Yes	23.5	---	14.6	1.61 (0.23, 11.2) ^a^	51.2	1.68 (1.18, 2.39)
*Time to toilet < 3 min away*						
No	27.8	1.0	22.9	1.0	42.9	1.0
Yes	16.1	0.86 (0.68, 1.08)	11.9	0.52 (0.21, 1.31) ^a^	46.8	1.07 (0.74, 1.55)
*No cooling measures available at current workplace*						
No	18.3	1.0	10.5	1.0	54.2	1.0
Yes	25.0	1.09 (0.88, 1.35)	23.1	1.16 (0.96, 1.41)	32.5	0.68 (0.48, 0.96)
*No use cooling measures used past week*						
No	16.4	1.0	15.9	1.0	50.0	1.0
Yes	33.3	1.25 (0.94, 1.67)	14.8	0.99 (0.82, 1.19)	33.3	0.75 (0.53, 1.07)
*Ever experienced a heat-related illness*						
No	15.3^b^	1.0	7.3^b^	1.0	50.7	1.0
Yes	37.0	1.35 (0.99, 1.83)	34.6	1.42 (1.06, 1.89)	33.3	0.74 (0.52, 1.06)
*Experienced HRI past week*						
No	38.1	1.0	25.0	1.0	0.0	1.0
Yes	33.3	0.93 (0.48, 1.79)	66.7	2.25 (0.71, 7.17)	42.9	---
*High heat knowledge score*						
No	---		10.7 ^b^	1.0	50.0	1.0
Yes	---		33.3	1.34 (0.98 1.83)	28.6	0.70 (0.49, 0.99)
*Very concerned about HRI*						
No	17.3 ^b^	1.0	---		48.8	1.0
Yes	46.7	1.55 (0.96, 2.52)	---		33.3	0.77 (0.51, 1.17)
*Very comfortable taking a break*						
No	27.8^ b^	1.0	19.6	1.0	---	
Yes	13.3	0.48 (0.20, 1.13) ^a^	11.4	0.58 (0.21, 1.57) ^a^	---	

Notes: PR, prevalence ratio; ^**a**^ initial log-binomial model did not converge—95% CI based on exact confidence interval; **^b^** Chi-square or Fisher’s exact test *p*-value < 0.05.

## 4. Discussion

Results from this study are the first among farmworkers in Oregon, a large agricultural state but one not typically associated with HRI given the typical lower heat index values during the growing season than other parts of the country. One of the key findings from this study is that nearly 30% of participants reported experiencing two or more HRI symptoms in the previous week. Also, while 73% of workers reported drinking water at least once per hour the previous week, 65% of workers also acknowledged drinking soda at work, which is not recommended by the U.S. Occupational Safety and Health Administration (OSHA). Oregon does not have regulations addressing heat exposure among outdoor workers including farmworkers; however, OSHA recommends water, rest, and shade for workers working in hot conditions. While nearly 90% of participants’ employers provided fluids, 40% of participants reported that their employers did not provide cooling measures such as shade structures, trees, fans, rest stations, *etc*. Study results also show a low level of knowledge about working in the heat (21.0% scored high on heat knowledge index) as well as a low level of concern about working in the heat (only 15.6% were “very concerned” about risk of HRI at work). The average daily maximum temperature is 82 °F and 83 °F in July and August, respectively, with little humidity, which may account for the low knowledge and concern about working in hot conditions. However, all workers working in hot conditions are at risk for HRI. Additional information is needed about the content and frequency of training regarding HRI among farmworkers to better understand the low heat knowledge score. Finally, while workers paid by the piece were more likely to have a high heat knowledge score and be “very concerned” about HRI compared to workers paid by the hour, they were less likely to work in an environment in which they felt “very comfortable” taking a break. Additional research is needed to compare the working conditions and risk factors for HRI between workers paid by the piece and hour.

A recent study of farmworkers in California assessed heat-related knowledge and practices including a heat knowledge score, comfort level taking breaks, and level of concern regarding risk of HRI at work [10]. Stoecklin-Marois *et al*. found that 91.6% of farmworkers received training about HRI, 70% of workers scored high on the heat knowledge score, 79% reported being “very comfortable” taking breaks at work, and 8.7% were “very concerned” regarding risk of HRI at work. The high levels of training, knowledge and comfort level taking breaks as well as lower concern level regarding HRI are not surprising given California is one of two states which have adopted state-based standards specifically addressing HRI prevention in the absence of a federal occupational standard [13]. California’s standard requires employers to provide HRI training, water, shade and regular breaks as well as to have an emergency response plan in place.

Another recent study of 405 farmworkers in Georgia also assessed HRI symptoms, and knowledge and practices regarding HRI prevention [14]. Fleischer *et al*. found that over 50% of respondents reported experiencing two or more HRI symptoms in the previous week compared to 28% in the present study. The difference in number of HRI symptoms experienced is most likely the result of workers in Georgia working in conditions with higher heat index values. Fleischer *et al*. also found that over 53% of workers consumed soda during hot and humid conditions, contrary to OSHA recommendations, compared to 65% in the present study. Also, results from a 2010 study in North Carolina show that 40% of workers working in extreme heat experienced symptoms of HRI in their lifetime [8]. The present study did not assess lifetime symptoms of HRI; however, we found that 64% of workers experienced at least one HRI symptom in the previous week. The north Carolina study did not include heavy sweating, which 50% of respondents in the present study reported.

The present study has several limitations which should be noted. First, the study included a convenience sample of workers from four migrant camps in one county in Oregon. Working conditions were similar among workers at individual camps and the results, therefore, have limited generalizability. Therefore, we caution the use these results in making large changes in policy. Next, all data were self-reported and may suffer from recall error. However, any recall bias most likely led to non-differential misclassification resulting in underestimates of associations between heat knowledge, level of concern about HRI, and comfort level taking breaks and work and demographic characteristics (Table 4). Also, we did not collect data on environmental conditions (*i.e.*, heat index values) at the camps before and during data collection. Finally, HRI symptoms are non-specific and can be associated with other ailments. However, to increase specificity, participants were asked if they experienced symptoms during the previous week while working on a hot day. 

## 5. Conclusions

The study’s results demonstrate a need for more data on the working conditions, and knowledge and practices regarding HRI among farmworkers, not only in Oregon but throughout the Northwest, a region that is not typically recognized as high risk for HRI given its lack of humidity and resulting lower heat index values during the growing season. Additional research is also needed to prospectively determine the incidence of HRI during a growing season among a large sample of workers at multiple sites. This research should also measure environmental conditions and work conditions which predict HRI. The results also suggest several areas in which employers and agencies conducting outreach and education to the workers about HRI can change their practices including providing cooling measures and HRI training about risk factors for HRI. Oregon is geographically located between the only two states in the U.S. which have adopted state-based standards specifically addressing HRI prevention and is, therefore, uniquely positioned to compare the morbidity and mortality associated with working in hot conditions among farmworkers in the three states. 
